# Acute Gastric Volvulus on Hiatal Hernia

**DOI:** 10.1155/2020/4141729

**Published:** 2020-12-09

**Authors:** Abdessamad EL KAOUKABI, Mohamed MENFAA, Samir HASBI, Fouad SAKIT, Abdelkrim CHOHO

**Affiliations:** General Surgery, Moulay Ismail Military Hospital, Morocco

## Abstract

The gastric volvulus is defined as an abnormal rotation of all or part of the stomach around one of its axes, creating the conditions of an upper abdominal obstruction with gastric dilation and risk of strangulation. It is a rare entity that requires a surgical treatment, and its diagnosis is often delayed due to frequently aspecific symptoms. We will describe the observation of a 62 year old patient who presented to the emergency department for acute epigastric pain with dyspnea. The thoracoabdominal CT has demonstrated a stasis stomach on pyloric obstacle evoking a gastric torsion. An upper gastrointestinal endoscopy (EGD) and an upper gastrointestinal contrast made it possible to diagnose an acute gastric volvulus on hiatal hernia. A midline laparotomy was performed with detorsion of the stomach and repair of the hiatal hernia. The patient recovered gradually and was discharged on the sixth postoperative day. Three months after the operation, the patient remained asymptomatic.

## 1. Introduction

Gastric volvulus was first described by Ambroise Pare in 1957 in a patient with traumatic diaphragmatic rupture. It was only in the 19th century that this pathology began to be well individualized, in particular thanks to the work of Berti [1]. At the beginning of the 20th century, several definitions were proposed based on the rotation angle of the stomach and on etiological factors [2]. Thus, the gastric volvulus is defined by an abnormal rotation of all or part of the stomach around one of its axes, creating the conditions of an upper abdominal obstruction with gastric dilation and risk of strangulation. Considering the rotation axis, we will distinguish the organoaxial volvulus and the mesenteroaxial volvulus.

It is a rare disease, often underdiagnosed and unrecognized, which can lead to serious complications especially strangulation with ischemia and gastric necrosis. This indicates the importance of early diagnosis, which itself depends on better knowledge of this pathology in all its aspects.

We report here the observation of an acute gastric volvulus on hiatal hernia which was treated by surgery.

## 2. Presentation of Case

**Figure 1:**
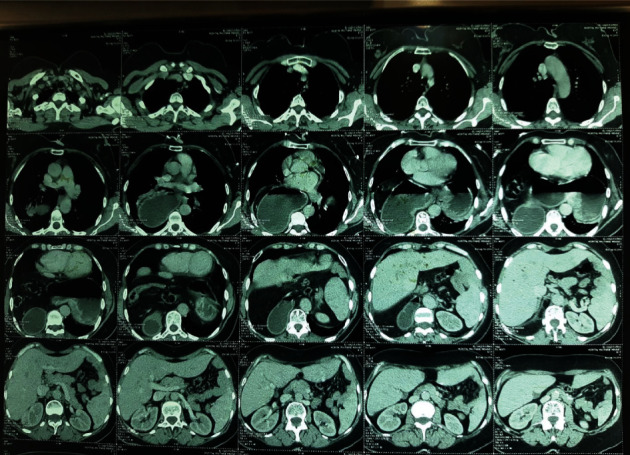
A thoracoabdominal CT scan showing a stomach stasis on a pyloric obstacle.

**Figure 2:**
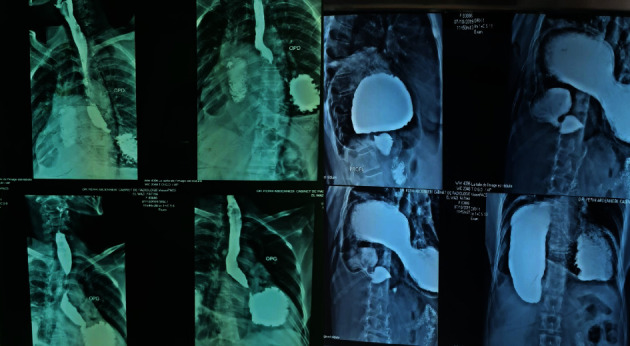
An upper gastrointestinal contrast showing a gastric plication with anteropyloroduodenal malrotation.

The patient was a 56-year old woman with type 2 diabetes on oral antidiabetic drugs, with a chronic history of epigastric pain and heaviness relieved by early postprandial vomiting, who has worsened symptomatology for 48 hours with epigastric pain and dyspnea. Physical examination found pulse was 100 beats/min and regular, BP 140/90 mm of Hg, respiratory rate 26 breaths/min, and temperature 37°C. Her abdomen was distended, and tenderness was present in the epigastric region by superficial and deep palpation. There was no organomegaly or any other palpable mass. Per rectal examination revealed no significant abnormality. The remainder of her lab results, including renal and liver function tests, was normal apart from a normochromic normocytic anemia (Hb = 9 g/dl). A thoracoabdominal CT scan was performed showing a stomach stasis on a pyloric obstacle suggesting stomach torsion (Figure 1); then, an upper gastrointestinal endoscopy was done showing a large hiatal hernia deforming the stomach with difficult antral passage. In front of clinical stability and the absence of complication signs, we allowed ourselves to make an upper gastrointestinal contrast which confirmed the diagnosis of gastric volvulus by showing a gastric plication with anteropyloroduodenal malrotation (Figure 2).

**Figure 3:**
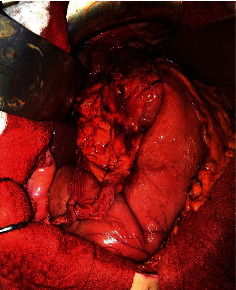
Closing of the hiatal orifice and performing a Toupet fundoplication.

The patient was referred to the operating room for surgery. Under general anesthesia, a midline laparotomy was performed; the exploration found an organoaxial gastric volvulus on mixed hiatal hernia without suffering signs. After detorsion and reintegration of the stomach, the hernial sac was partially resected, the hiatal opening was closed by separate stitches with nonabsorbable thread, and then a Toupet fundoplication was done (Figure 3). The patient recovered gradually and was discharged on the sixth postoperative day. Three months after the operation, the patient remained asymptomatic.

## 3. Discussion

It is a rare disease, often underdiagnosed and unrecognized, which can lead to serious complications especially strangulation with ischemia and gastric necrosis. This indicates the importance of early diagnosis, which itself depends on better knowledge of this pathology in all its aspects. This condition mainly affects the elderly with a peak frequency around the age of fifty [3]. Cases of young adults have been reported in which the etiopathogenesis was dominated by traumatic diaphragmatic lesions [4, 5]. Regarding gender, there is no predilection according to some authors [6], while others report a slight predominance of women [3, 7].

Several anatomopathological classifications have been proposed, and the most complete one is that proposed by Von Haberer and Singleton modified by Carter in 1978 [8] who described 3 types of gastric volvulus according to the rotation axis. Organoaxial volvulus is a rotation of the stomach around a longitudinal axis passing through the cardia and the pylorus. It is the most common form, occurring in approximately 60% of cases [4]. In mesenteroaxial volvulus, which comprises 29% of cases, rotation occurs along a transverse, medigastric axis, passing through the midpoints of the small and the great curvature [9]. And finally, the last type is the combination-unclassified volvulus.

Hiatal hernias are the number one cause of gastric volvulus. The incarceration of the stomach is favored by a negative intrathoracic suction pressure. Rolling hiatal hernias represent 50% of the causes of gastric volvulus in adults, unlike sliding forms which are exceptionally at the origin of this pathology [6, 10]. However, it should be noted that only 4% of hiatal hernias are complicated by gastric volvulus, signifying the rarity of this pathology [11]. In our case, we were dealing with a gastric volvulus on mixed hiatal hernia. The other types of diaphragmatic hernias, splenic and hepatic anomalies, complete the main etiologies of gastric volvulus.

Clinical picture depends on the rotation degree, gastric obstruction, and sub- or supradiaphragmatic position; 3 types of presentation can be described.

Chronic gastric volvulus affects 30% of gastric volvulus [12]. Symptoms are dominated by abdominal pain, type of gastric heaviness or oppression, often after meals, relieved by vomiting and accompanied by early satiety [13].

The intermittent or subacute gastric volvulus evolves by crises yielding spontaneously and suddenly. It is the prerogative of partial volvulus of mesenteroaxial type. The functional signs are often related to gastric emptying difficulties. Cardiopulmonary signs may be present. And the acute gastric volvulus is often the prerogative of complete volvulus and occurs especially in the elderly [9]. The famous Borchardt triad, which includes severe epigastric pain with distention, vomiting followed by violent, nonproductive retching, and finally difficulty or inability to pass a nasogastric tube into the stomach, is highly suggestive of the diagnosis [14]. However, the concomitant presence of these 3 signs is rare and we will retain the picture of a sudden upper intestinal obstruction [8]. In our case, it was the sudden onset dyspnea in a patient with a chronic history of digestive symptoms that made up the bulk of the clinical picture.

To establish a positive diagnosis of gastric volvulus, the upper gastrointestinal contrast study is the reference examination. It is performed in the absence of cardiorespiratory shock, peritonitis, or mediastinitis where the surgical indication cannot be discussed [3, 15]. Due to the rarity of this disease, the contribution of computed tomography is currently not well established although this examination is of great interest in the positive diagnosis allowing specification of the type of volvulus, its cause, and especially the cardiopulmonary consequences of intrathoracic migration [16]. Upper gastrointestinal endoscopy is not of great diagnostic interest but above all makes it possible to search for an etiology such as a hiatal hernia and to study the gastric mucosa state. It is contraindicated if gastric necrosis or perforation signs are present. Unfortunately, this exploration is often incomplete due to the gastric torsion which prevents the progression of the fibroscope [17].

The initial management for acute gastric volvulus includes resuscitation, placement of a nasogastric tube, gastric decompression, and resting in the prone position [18]. Most authors agree on the principle that emergency or deferred emergency surgical treatment is essential even in chronic forms that are long asymptomatic because of the permanent and unpredictable risk of strangulation [19]. The aim of the surgical procedure is to restore the normal anatomical position of the stomach and to prevent further episodes by correcting the predisposing factors with or without gastropexy [20, 21]. In our case, we chose to perform a Toupet fundoplication since the patient reported a chronic history of digestive symptoms probably related to a possible reflux.

In the event of complications such as gangrene and perforation, gastrectomy or partial gastrectomy should be performed. The approach to surgery can be performed by open and laparoscopic techniques [22–24]. The laparotomy is the most used especially in case of acute volvulus allowing broad access to the abdominal cavity [3, 25]. Laparoscopic surgery has largely demonstrated its usefulness in elective surgery for chronic gastric volvulus but also in some cases of acute volvulus. Koger and Stone [26], in 1993, achieved the first laparoscopic success in the treatment of acute gastric volvulus by performing reduction and gastropexy. After devolvulation and reintegration of the stomach, treatment of predisposing factors in cases of secondary gastric volvulus is necessary to prevent recurrences. Untwisting the stomach and repair of secondary defects such as hiatus hernia have been treated successfully using a laparoscopic approach. Performing antireflux surgery in the same sitting, when repairing the paraesophageal defect, may be attempted [23, 24]. In a case series of 29 patients who presented with acute gastric volvulus, 13 underwent laparoscopic surgery with 2 conversions to open surgery, another 13 underwent open surgery, and 3 were treated conservatively. None of the patients had any major complications [22]. Other less invasive methods can be attempted for patients with acute gastric volvulus, especially those who are at high surgical risk due to their comorbidities. One of these methods is the use of dual endoscopy in the form of OGD to untwist the volvulus, with fixation with a PEG tube [27]. However, performing gastropexy endoscopically using a PEG tube has its own limitations due to the risk of recurrence consequent to inadequate fixation, persistence of predisposing factors such as hernias and adhesions from other surgeries, and the potential that the fixation point will act as an axis for further rotations [28].

## 4. Conclusion

Gastric volvulus is a rare disease which should be considered a diagnostic and therapeutic emergency due to the risk of gastric necrosis by prolonged ischemia which can be life-threatening.

We should always think of gastric volvulus as a differential diagnosis in case of acute epigastric pain associated with vomiting or dyspnea especially with history of diaphragmatic hernia or chronic digestive symptoms.

The treatment of choice is always immediate surgical intervention combining devolvulation, reintegration of the stomach, and the treatment of the cause.
